# In Vitro Evaluation of Confounders in Brain Optical Monitoring: A Review

**DOI:** 10.3390/s25185654

**Published:** 2025-09-10

**Authors:** Karina Awad-Pérez, Maria Roldan, Panicos A. Kyriacou

**Affiliations:** 1 Research Centre for Biomedical Engineering, City St George’s University of London, London EC1V 0HB, UK; p.kyriacou@citystgeorges.ac.uk; 2 Crainio Limited, London SE10 8QQ, UK

**Keywords:** optical brain monitoring, phantom, confounders, skin pigmentation, skull thickness, extracerebral layers, brain pathologies

## Abstract

**Highlights:**

**What are the main findings?**

- In vitro studies using head phantoms show that confounders such as extracerebral layers, skin pigmentation, and skull thickness affect the accuracy of parameters estimated using optical technologies.
- Existing head phantom models often oversimplify anatomical structures and lack the ability to simulate physiological conditions like pulsatile flow.

**What are the implications of the main findings?**

- Further research is needed to isolate and quantify the effect of individual confounders on the optical signal, beyond device-specific outputs.
- Developing head phantoms capable of generating pulsatile signals is essential to evaluate PPG-based cerebral monitoring systems.

**Abstract:**

Optical brain monitoring techniques, including near-infrared spectroscopy (NIRS), diffuse correlation spectroscopy (DCS), and photoplethysmography (PPG) have gained attention for their non-invasive, affordable, and portable nature. These methods offer real-time insights into cerebral parameters like cerebral blood flow (CBF), intracranial pressure (ICP), and oxygenation. However, confounding factors like extracerebral layers, skin pigmentation, skull thickness, and brain-related pathologies may affect measurement accuracy. This review examines the potential impact of confounders, focusing on in vitro studies that use phantoms to simulate human head properties under controlled conditions. A systematic search identified six studies on extracerebral layers, two on skin pigmentation, two on skull thickness, and four on brain pathologies. While variation in phantom designs and optical devices limits comparability, findings suggest that the extracerebral layer and skull thickness influence measurement accuracy, and skin pigmentation introduces bias. Pathologies like oedema and haematomas affect the optical signal, though their influence on parameter estimation remains inconclusive. This review highlights limitations in current research and identifies areas for future investigation, including the need for improved brain phantoms capable of simulating pulsatile signals to assess the impact of confounders on PPG systems, given the growing interest in PPG-based cerebral monitoring. Addressing these challenges will improve the reliability of optical monitoring technologies.

## 1. Introduction

The main objective of this review is to synthesise existing in vitro approaches used to evaluate the impact of confounding factors on optical brain monitoring. In particular, it examines how different phantom designs and experimental setups have been utilised to isolate the effects of variables such as extracerebral layers, skin pigmentation, skull thickness, and brain-related pathological conditions, while highlighting their strengths and limitations to provide suggestions for future research.

Though the influence of confounders in brain optical monitoring is recognised, most existing evidence is either theoretical or derived from simulations. What remains unclear is the extent to which each variable quantitatively affects light-tissue interaction under controlled settings. Researchers have conducted in vivo studies and clinical trials to investigate the practical implications of these confounding factors. However, these approaches face significant challenges. In vivo studies are expensive, requiring substantial financial resources and extended research timelines due to regulatory approvals and ethical clearances [1,2]. Moreover, in studies conducted with healthy volunteers, physiological changes such as hypoxia can only be induced within safe limits, preventing the investigation of extreme or pathological conditions [3,4,5]. Ethical concerns also arise when invasive procedures, such as jugular venous sampling for oximetry validation, are performed on healthy subjects, as these carry risks of infection or bleeding [1].

Given these limitations and the growing presence of these technologies in the market, there is a need for controlled experimental approaches using tissue-simulating phantoms. Phantoms provide a standardised platform for evaluating the effects of confounders in a reproducible manner, eliminating the ethical and logistical constraints associated with in vivo studies. By mimicking the optical properties of biological tissues, phantoms enable researchers to investigate the effect of specific variables.

Therefore, this review critically evaluates the ways in which in vitro studies have employed head phantoms and controlled experimental setups to investigate the impact of individual confounders on optical brain monitoring. By assessing the strengths and limitations of prior research, it aims to guide future work towards the development of more physiologically realistic phantom models and a deeper understanding of the mechanisms through which confounders influence the optical signal itself. Ultimately, the goal is to support the advancement of more accurate, reliable, and clinically applicable optical brain monitoring technologies.

## 2. Background

Optical brain monitoring has gained popularity due to its advantages over traditional methods such as computed tomography (CT), magnetic resonance imaging (MRI), and invasive intracranial probes. Optical techniques are non-invasive, provide rapid diagnosis, and enable timely clinical decisions that improve patient outcomes [6,7]. They are more affordable and accessible than invasive alternatives, making them particularly valuable in low-resource settings [6,7], and their portability allows for bedside monitoring and use in remote locations. Another advantage is their potential for multimodal monitoring, in which multiple physiological parameters can be extracted from a single sensor [6,7].

These techniques rely on the interaction of light with biological tissues, primarily using near-infrared wavelengths, which can penetrate deeper into head tissues [8]. Measurements of the absorption and scattering of light by chromophores in the brain allow for the estimation of physiological parameters, such as cerebral blood flow (CBF) [9], intracranial pressure (ICP) [5], cerebral oxygenation [8], among others. The most commonly used optical techniques include near-infrared spectroscopy (NIRS), which measures changes in haemoglobin concentration or oxygenation indices, and diffuse correlation spectroscopy (DCS), which measures CBF [10]. DCS is often combined with NIRS for a more comprehensive haemodynamic assessment [10]. Additionally, photoplethysmography (PPG) has been explored for cerebral monitoring. While traditionally used in vascular assessments, cerebral PPG is being investigated for its potential to assess brain health, as intracranial hypertension affects flow pulsatility and may influence PPG waveform characteristics [11]. Theoretically, the DC component of cerebral PPG could also be used to track changes in haemoglobin levels.

Despite their current use and potential, optical brain monitoring techniques are susceptible to confounding variables that can negatively impact measurements and compromise their accuracy. These confounders are intrinsic to the patient and cannot be controlled. For instance, extracranial layers, including the scalp and its vasculature, can absorb and scatter light, reducing penetration depth [12,13,14]. The extracranial vasculature may also mislead estimations, particularly when the scalp and brain have different oxygenation levels [8]. Skin pigmentation has also been identified as a potential source of bias in optical measurements, as melanin affects skin optical properties and may alter readings [15,16,17]. Additionally, skull thickness is a relevant confounding factor, as skull dimensions vary across populations, particularly between men and women and across different age groups [18]. This could partially explain sex-based differences in oximetry readings [15]. Finally, intracranial pathologies such as oedema, haematomas, and haemorrhages can further complicate optical monitoring. Brain swelling can also alter photon pathlength and increase intracranial scattering, while haematomas and haemorrhages may absorb significant amounts of light, limiting signal transmission [8].

Several companies have developed, or are actively developing, optical brain monitoring technologies for clinical or research use. For example, the Root O3 Regional Oximeter (Masimo Corporation, Irvine, CA, USA), the NIRO series (Hamamatsu Photonics, Hamamatsu City, Japan), and the INVOS system (Medtronic, Minneapolis, MN, USA) are widely used for cerebral oximetry in perioperative care and research settings. In addition, startups such as Cranio (London, UK) and Wavewise Analytics (Melbourne, Australia) are exploring innovative optical approaches, including cerebral PPG, for non-invasive brain monitoring [5,19].

## 3. Materials and Methods

To identify studies evaluating confounding variables in brain optical monitoring using in vitro approaches, a systematic search was conducted in Scopus, PubMed, and IEEE Xplore between 28 November and 3 December 2024. The search strategy employed a combination of keywords across all metadata, including terms related to in vitro studies, optical techniques (e.g., “NIRS,” “DCS,” “PPG,” and their variations), and specific confounders such as skin pigmentation and skull thickness (Table 1). Studies were included regardless of publication year. Studies without full-text availability in English were excluded. This literature review follows a rigorous search strategy, yet quality assessment of the papers included in the synthesis was not conducted.

Following the removal of duplicates, studies were screened by title and abstract to determine their eligibility. Studies were included if they focused on brain monitoring, employed an in vitro approach to assess a confounder using optical techniques, were published in English, and had full texts available. Conference proceedings were also considered eligible for inclusion.

The selected studies were categorised into four groups based on the confounder assessed: extracerebral layers, skin pigmentation, skull thickness or concomitant pathologies. A qualitative synthesis was performed in which each study was summarised, and key information was extracted, including the confounder investigated, the optical technique used, the main characteristics of the phantom models, and the study’s main findings. Thematic comparison related to each confounding factor was carried out to highlight trends, discrepancies, and gaps in current knowledge.

## 4. Results

### 4.1. Study Selection

**Figure 1:**
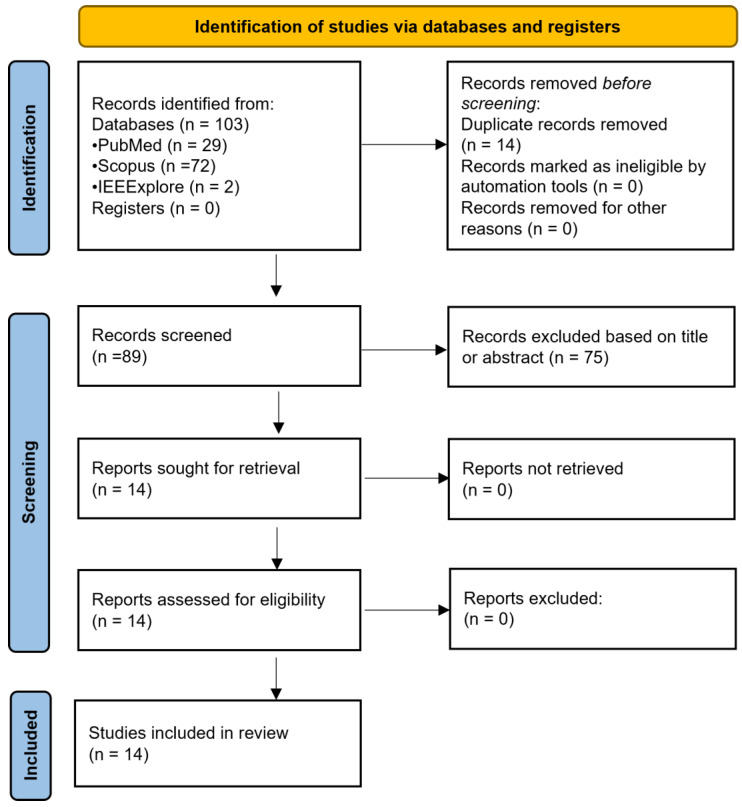
Flow diagram illustrating the systematic search process, from Preferred Reporting Items for Systematic Reviews and Meta-Analyses (PRISMA). *n* = number of studies.

From an initial total of 103 papers, 14 duplicates were removed, and 75 additional papers were excluded during title and abstract screening as they were unrelated to the topic or did not assess the effect of a confounder in optical brain monitoring. The full texts of the remaining 14 studies were sought for retrieval, successfully obtained, and assessed for eligibility. As a result, all 14 papers were included in the final analysis. Figure 1 illustrates the process of identification, screening, and inclusion of papers.

### 4.2. Extracerebral Layers

Regarding the influence of extracerebral layers on optical monitoring, six studies were identified and categorised based on the optical technique used, either NIRS or DCS. None of the studies investigated the effects on PPG.

#### 4.2.1. Near-Infrared Spectroscopy (NIRS)

**Figure 2:**
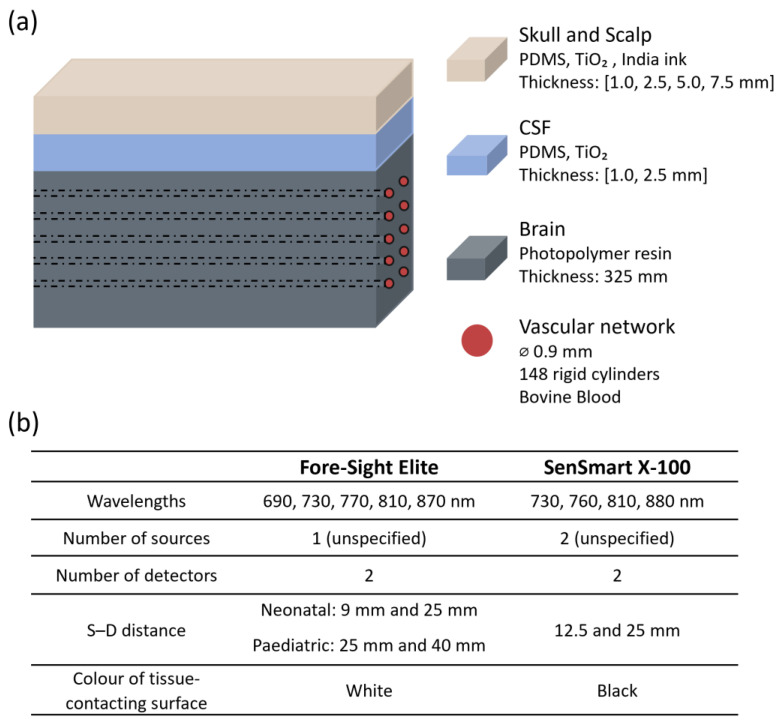
Representation from the study by Afshari et al. [3]. (**a**) Head phantom model showing layer materials and features; (**b**) sensor characteristics. PDMS = polydimethylsiloxane; TiO_2_ = titanium dioxide; CSF = cerebrospinal fluid; ⌀ = diameter; S–D: source–detector distance.

The effect of extracranial layer thickness on NIRS based cerebral oximetry was assessed by Afshari et al. in 2019 [3] using two commercial devices: Fore-Sight Elite (CAS Medical Systems, Inc., Branford, CT, USA) and SenSmart X-100 (Nonin Medical, Inc., Plymouth, MN, USA), with different sensors attached, as shown in Figure 2b. The extracerebral layer, where they combined skin, scalp, and skull as only one layer, was modelled with thicknesses of 1.0, 2.5, 5.0, and 7.5 mm. The layer optically matched the adult scalp and was made using a mixture of polydimethylsiloxane (PDMS), titanium dioxide (TiO_2_), and India ink. The study also incorporated a layer to simulate cerebrospinal fluid (CSF) made of PDMS and TiO_2_. The brain, optically similar to neonatal cerebral tissue, was developed using a solid phantom approach based on a 3D-printed cerebrovascular module incorporating an array of 148 cylindrical, non-pulsatile channels (Figure 2). These channels were filled with whole bovine blood, and sodium dithionite was used to induce desaturation. Oxygen saturation levels ranged from 30% to 90% and were measured using CO-oximetry.

The research found that increasing the thickness of the superficial layer consistently reduced both accuracy and sensitivity for the Fore-Sight Elite device. Errors were particularly pronounced when using the neonatal sensor, which has a smaller source–detector separation. The root-mean-square error (Arms) increased from 5% with no superficial layer to 21% at the maximum modelled extracranial thickness. In contrast, the SenSmart device showed less variability in response to changes in layer thickness, maintaining an Arms below 10%. The authors attributed this difference to the sensors’ surface reflectivity. The tissue-contacting surface of the SenSmart sensor is black, which absorbs light and minimises the re-entry of reflected photons into the medium. In contrast, the Fore-Sight sensor surface is white and more reflective, enabling exiting photons to re-enter the tissue and propagate within the superficial regions, thereby increasing the detection of shallow-penetrating light [3]. These findings highlight how probe design elements, such as source–detector distance, wavelength selection, and the optical properties of the sensor surface, can influence the impact of confounders.

**Figure 3:**
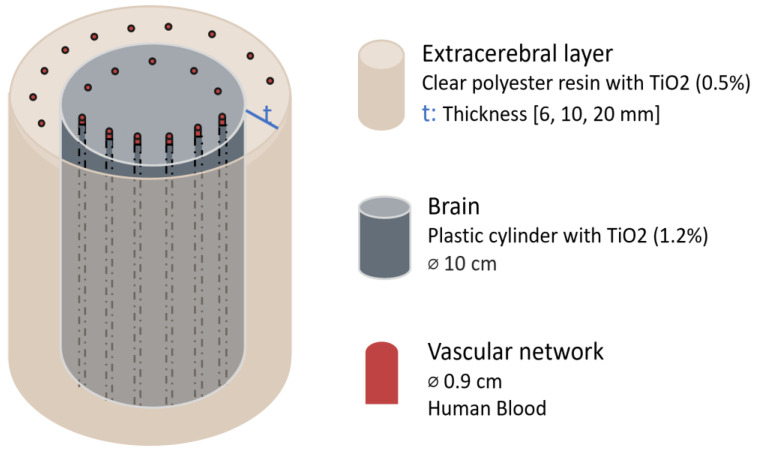
Head phantom representation from the study by Kurth & Thayer [20]. Figure illustrates each element material and dimension. The extracerebral layer thickness was varied. TiO_2_ = titanium dioxide; ⌀ = diameter.

A different study, conducted by Kurth and Thayer in 1999, investigated whether thickness and oxygenation levels of extracerebral layers could confound brain oxygen saturation (SO_2_) estimates using a multiwavelength frequency-domain NIR oximeter (fdNIRS) (PMD 4002, NIM Incorporated, Philadelphia, PA, USA) in an in vitro brain model [20]. The model, which simulated the optical properties of the piglet brain, consisted of a brain phantom surrounded by a series of shells designed to simulate extracranial tissues of infants, children, and adults. The brain phantom was a 10 cm diameter plastic cylinder containing a microvascular network (0.9 mm). Surrounding it, shells of varying thickness (6 mm, 10 mm, and 20 mm) were constructed from clear polyester resin and had their own vascular networks embedded. To mimic optical scattering properties, a TiO_2_ emulsion was added at concentrations of 1.2% for the brain and 0.5% for the surrounding layer, simulating the lower scattering properties of the skull. The system was perfused with human blood. Blood SO_2_ was regulated by adjusting the flow of O_2_ and N_2_ in the circuit and measured using co-oximetry. To assess the effect of extracranial layers, fdNIRS SO_2_ measurements were recorded while the phantom brain was perfused with either oxygenated or deoxygenated blood, with the extracerebral layer containing blood of the opposite oxygenation state or remaining empty. Figure 3 shows a representation of this head phantom.

The results demonstrated that extracerebral thickness influenced the accuracy of fdNIRS SO_2_ readings. For thinner extracerebral layers, such as 6 mm (infant) and 10 mm (children) the fdNIRS measurements remained unaffected. However, for a 20 mm thickness, representing adult extracranial tissues, there were inaccuracies: under conditions representing an oxygenated brain, the presence of an extracerebral shell containing either no blood or deoxygenated blood led to a decrease in measured SO_2_ by 26 ± 3% and 32 ± 6%, respectively. Conversely, when modelling a deoxygenated brain, extracerebral shells without blood or with oxygenated blood caused an overestimation of SO_2_ by 21 ± 7% and 26 ± 3%, respectively.

**Figure 4:**
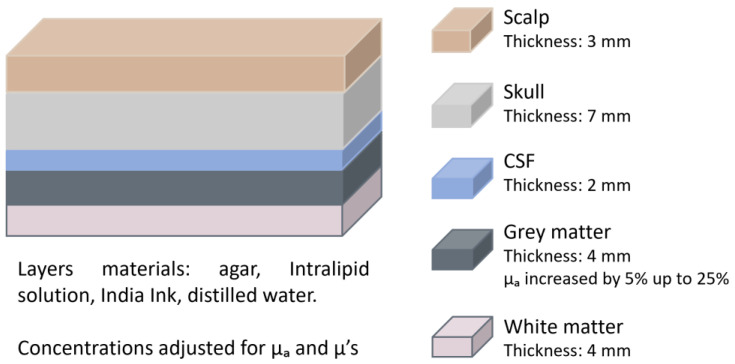
Head phantom representation from the study by Zhang et al. [21]. The figure depicts each layer with its corresponding thickness. The optical properties of the grey matter were varied. CSF = cerebrospinal fluid; μa = absorption coefficient; μ’s = reduced scattering coefficient.

In 2019, Zhang et al. [21] investigated the influence of extracerebral layers for optical properties measurement using continuous-wave NIRS (CW-NIRS). They developed a five-layer phantom that mimicked the scalp, skull, CSF, grey matter, and white matter (Figure 4). The layers were composed of agar, Intralipid solution, and India Ink, with optical properties customised by adjusting the concentrations of absorber and scatterer to match those of pig skull as well as human brain and dermis. To simulate brain activity, the absorption coefficient (μa) of the grey matter layer was varied by up to 25% in 5% increments. The NIRS system was used to track the changes in the grey matter’s absorption. The thickness of the extracerebral layers was kept constant, as the objective was to evaluate whether their mere presence affected measurement accuracy. Results showed that the recorded changes in μa were consistently smaller than the actual variations, indicating contamination by superficial layers. The authors proposed Monte Carlo simulations as a potential correction method, assuming that the anatomical structure of the brain is determined by other methods.

#### 4.2.2. DCS

**Figure 5:**
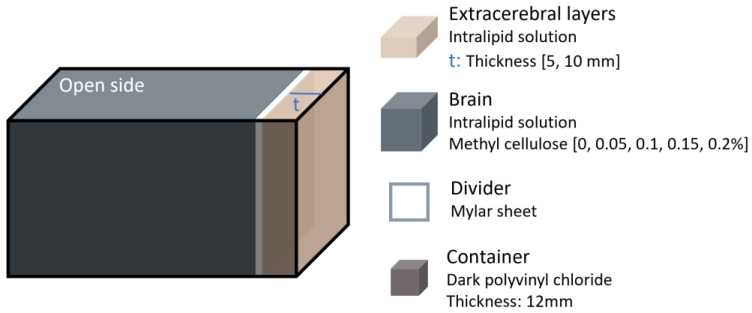
Head phantom representation from the study performed in 2016 by Verdecchia et al. [22]. The extracerebral layer thickness and brain viscosity were varied.

More complex systems, such as DCS and hybrid systems that combine multiple optical techniques, have also been used to evaluate the effects of confounders. For instance, in 2016 Verdecchia et al. [22] conducted phantom experiments to validate a method they developed for successfully separating scalp and brain flow in DCS data analysis. The experiment utilised a two-layered phantom designed to allow independent alteration of the flow properties in each layer (Figure 5). The phantom consisted of a box made of dark polyvinyl chloride, with a polyester Mylar sheet inserted to separate the top and bottom layer. This sheet was positioned at either 5 mm or 10 mm from the top of the box. Both layers were filled with a 0.8% Intralipid solution to mimic tissue scattering. The viscosity of the bottom layer was increased by adding methyl cellulose at varying concentrations (ranging from 0% to 0.2%), whereas the top layer viscosity remained constant. The optical properties of the phantom were not directly compared to human tissue. Data acquisition was performed using a DCS instrument with a continuous-wave laser emitting at 785 nm and two source–detector distances (20 and 30 mm).

The results showed that the proposed model successfully resolved differences in flow rates between the two layers. However, the error in the measured diffusion coefficient increased with top layer thickness, reaching up to ~40% at 10 mm thickness.

**Figure 6:**
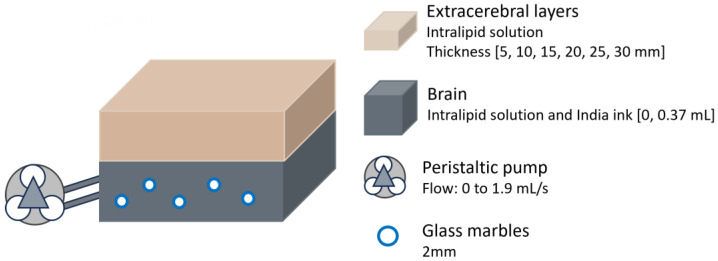
Head phantom representation from the study performed in 2015 by Verdecchia et al. [23]. The bottom layer had variable absorption and was connected to a peristaltic pump. Thickness of extracerebral layer varied.

In another study conducted in 2015, researchers assessed the depth sensitivity of a hybrid DCS/time-resolved (TR)-NIRS system at different source–detector distances to mitigate signal contamination from extracerebral tissues [23]. This hybrid approach would enable the simultaneous measurement of cerebral oxygenation using NIRS and blood flow using DCS. The experiment utilised a two-layer Intralipid phantom, with the bottom layer connected to a peristaltic pump to simulate cerebral blood flow (Figure 6). To randomise flow direction, 2 mm glass marbles were added (though it is unspecified whether this dimension corresponds to the radius or diameter). Flow in the bottom layer was varied from 0 to 1.9 mL/s, and its absorption was independently adjusted by adding India ink to mimic different haemoglobin concentrations in the brain, although a direct comparison to human brain values was not reported. The thickness of the top layer was incrementally increased in 5 mm steps up to 30 mm.

The results demonstrated that the impact of the extracerebral layer on estimated absorption and flow depended on source–detector distance. Depth sensitivity, which is the ability to detect signals from deeper cerebral tissues while minimising contamination from superficial layers, increased with greater source–detector separation. However, as the top layer thickened, the system’s ability to correct for extracerebral contamination decreased. For layers 20 mm thick or more, depth sensitivity dropped below 20%, leading to a significant underestimation of absorption changes in the bottom layer. Similarly, for flow detection, the system failed to capture variations in the deeper layer when the top layer exceeded 20 mm. Thinner top layers allowed for improved sensitivity to flow changes, though only when the source–detector distance was greater than 20 mm, highlighting the impact of probe design.

**Figure 7:**
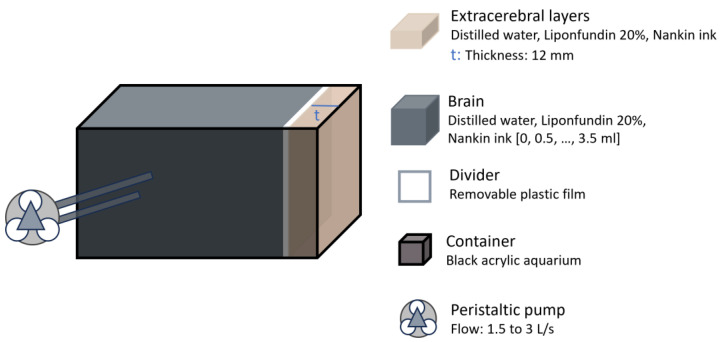
Head representation from the study by Forti et al. [24]. The brain layer absorption was adjusted using ink and flow was controlled using a peristaltic pump.

Similarly, in 2023 Forti et al. tested their algorithm for separating cerebral signals from extracerebral contamination using in vitro data collected with a hybrid DCS and frequency-domain diffuse optical spectroscopy (FD-DOS) system [24]. The in vitro setup consisted of a two-layered liquid phantom contained in a black acrylic aquarium filled with an Intralipid solution (Figure 7). A plastic film was attached to separate the layers, positioned 12 mm from the top. The absorption of the bottom layer was modified by adding ink, though a direct comparison to human brain tissue was not provided. This layer was connected to a peristaltic pump to vary flow after each ink addition. The algorithm demonstrated that flow indices estimated for the first layer remained relatively independent of absorption and flow changes in the second layer. Flow measurements in the second layer remained unaffected by variations in its μa. However, the authors were unable to estimate errors in flow recovery, as the pump flow in the phantom does not directly correspond to a true blood flow index.

Overall, there is consensus among studies that the thickness of extracerebral layers influences the accuracy of cerebral parameter estimates [3,20,22,23]. The extent of this effect depends on specific sensor characteristics, such as reflectivity [3] and source–detector distance [23]. Only one study suggested that the device was unaffected by flow changes in the second layer; however, the error was not quantified [24]. A summary of the studies analysing the effect of extracerebral layers is presented in Table 2, specifying the optical technique utilised, the main characteristics of the phantom, the primary outcome of the investigation, as well as the authors and publication year.

### 4.3. Skin Pigmentation

The impact of skin pigmentation on optical devices has been mainly studied in pulse oximetry for estimating peripheral oxygen saturation (SpO_2_). However, its role in brain monitoring remains largely unexplored. Only two in vitro studies were found that evaluated the effect of skin pigmentation on brain optical monitoring, both using commercial NIRS-based oximeters from different brands.

**Figure 8:**
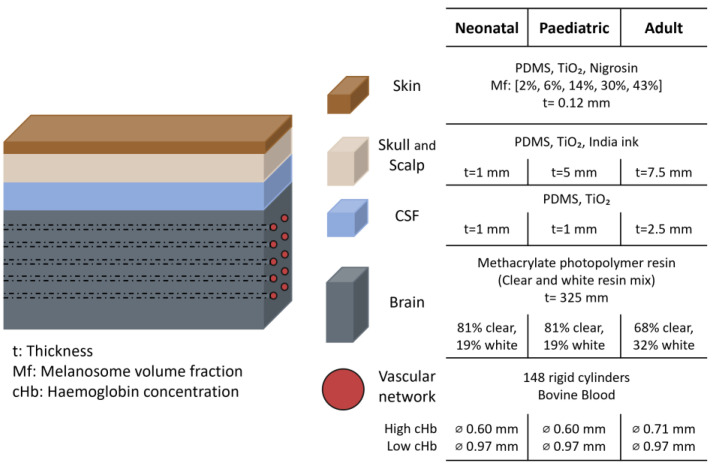
Head representation from the study by Afshari et al. [1]. The figure shows the materials and dimensions of each layer, highlighting differences between neonatal, paediatric, and adult phantom. PDMS = polydimethylsiloxane; TiO_2_ = titanium dioxide; CSF = cerebrospinal fluid; ⌀ = diameter.

In 2022, Afshari et al. expanded their previous phantom model [3] to assess the impact of skin pigmentation on NIRS-based commercial oximeter readings [1]. They used the same devices as in their previous study: Fore-Sight Elite (CAS Medical Systems, Inc., Branford, CT, USA) and SenSmart X-100 (Nonin Medical, Inc., Plymouth, MN, USA), with neonatal, paediatric, and adult sensors (sensors characteristics shown in Figure 2b) [16]. To create skin layers with varying pigmentation levels, synthetic melanin and different pigments were evaluated. Water-soluble nigrosine was identified as the pigment most accurately matching human melanin in the 600–900 nm range. Skin pigmentation levels were defined using the melanosome volume fraction (Mf), a measure of skin tone that is more objective than the Fitzpatrick scale or other self-reported methods [1]. Five levels of pigmentation were created by varying the nigrosine concentration, with Mf values of 2%, 6%, 14%, 30%, and 43%, corresponding approximately to Fitzpatrick skin phototypes I–I, III, IV, V, and VI, respectively. The μa and μ’s from the skin mimicking tissue was matched to values reported in the literature for human skin. The skin layers were approximately 0.12 mm thick. The thickness of the other layers varied depending on whether the setup simulated neonatal, paediatric, or adult anatomy, as shown in Figure 8. Similarly, the diameter of the embedded brain channels was adjusted to simulate different haemoglobin concentration (cHb) levels. The channels were filled with whole bovine blood, and sodium dithionite was added to induce progressive deoxygenation. Oxygen saturation levels, ranging from 30% to 90%, were quantified using CO-oximetry.

Measurements indicated that all device and sensor combinations were affected by epidermal pigmentation. Cerebral oximeter outputs consistently decreased with increasing simulated melanin content, with the effect being most pronounced at low saturation levels. This trend may be explained by melanin absorption, which is spectrally more similar to deoxyhaemoglobin than oxyhaemoglobin [1]. However, the degree to which melanin influenced the accuracy of oxygen estimation varied depending on the sensor’s design, particularly its tissue-contact surface reflectivity and source–detector distance.

Probes with highly reflective surfaces (e.g., white sensors like those used in Fore-Sight devices) were more sensitive to melanin, producing less accurate readings due to photons repeatedly crossing the epidermis [1]. The influence of pigmentation was most significant in neonatal sensors, likely due to the sensor’s higher reflectivity. Regarding source–detector distance, adult probes, which have larger distances, were less affected by skin pigmentation. Larger distances allowed for greater mean penetration depth of detected light, reducing interactions with the epidermis compared to the shorter-distance sensors used in neonatal and paediatric probes [1].

**Figure 9:**
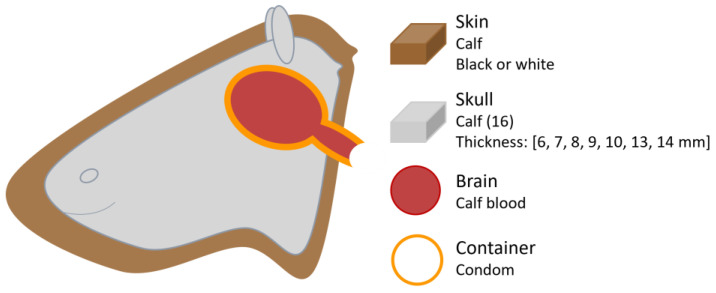
Head phantom representation from the study by Pringle et al. [25]. Figure illustrates the phantom components, and the varying thicknesses of the calf skulls used.

In a prior study conducted in 1998, Pringle et al. [25] combined in vitro and ex vivo methods to investigate factors influencing NIRS measurements. They utilised calf skulls from which the brain had been removed to accommodate a liquid phantom consisting of oxygenated calf blood dilutions contained within a condom (Figure 9). The blood’s partial pressure of oxygen was progressively reduced from above 110 mmHg to less than 10 mmHg using yeast and verified using a blood gas analyser (AVL 995, Graz, Austria). A NIRO-500 device (Hamamatsu Photonics, Hamamatsu-City, Japan) was used to measure haemoglobin changes and assess the influence of overlying calf skin colour on the NIRS signal. This device emits at wavelengths of 775, 827, 849, and 909 nm, and had a fixed source–detector separation of 40 mm.

The results indicated that no detectable difference in attenuation of the NIRS signal was observed between black and white skin. However, the presence of skin over the skull did cause attenuation compared to measurements taken with the bare skull. This finding relates more to the presence of extracranial layers rather than skin pigmentation itself. Nonetheless, Pringle et al. [25] noted that their study design did not allow sufficient focus on skin colouration as a factor.

The findings of both studies regarding skin pigmentation are contrasting: one study suggested that skin pigmentation does not significantly affect measurements [25], while the other reported a clear influence, heavily dependent on sensor reflectivity and source–detector distance [1]. A summary of the two studies reviewed in this section can be found in Table 3, including publication details, the optical technique used, phantom characteristics and key findings.

### 4.4. Skull Thickness

Two studies investigated the influence of skull thickness, both utilising NIRS commercial oximeters from different manufacturers. In Pringle et al. study [25], described above (Figure 9), the influence of skull thickness was evaluated using 16 calf heads with varying thicknesses. All except the two thickest skulls (13 and 14 mm) allowed sufficiently strong NIRS signals to accurately detect changes in haemoglobin oxygenation.

**Figure 10:**
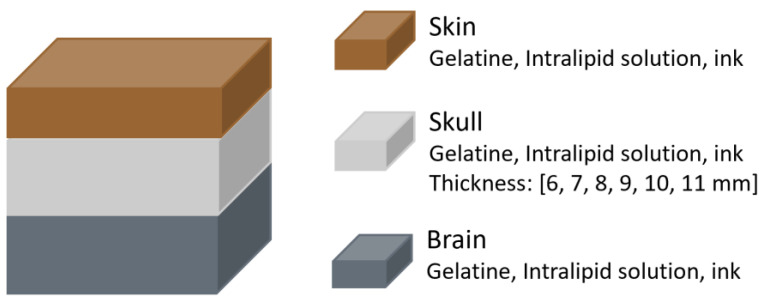
Head phantom representation from the study by Leibuss et al. [26]. The materials of each layer are shown, with the skull layer having variable thickness.

In 2021, Leibuss et al. [26] investigated whether skull thickness impacts regional cerebral oximetry (rSO_2_) measurements using the INVOS 5100C (Medtronic Limited, Watford, UK). This device uses two photodiodes that emit light at 730 and 810 nm, and two photodetectors positioned at distances of 30 and 40 mm from the source. To replicate the skull, phantoms were created using a combination of gelatine, an Intralipid water solution (acting as a scatterer), and ink (acting as an absorber), with concentrations adjusted to match the optical coefficients of pig skull. Six skull phantoms were prepared, with thicknesses ranging from 6 to 11 mm. Brain phantoms were fabricated using the same materials (Figure 10). A skin layer was also included, made with the same components, although its thickness was not reported.

The results indicated that skull thickness influenced regional oxygen saturation (rSO_2_) measurements. The difference between rSO_2_ values from the brain alone and from the brain with overlying skull and skin decreased as skull thickness increased. For example, a 6 mm skull resulted in a difference of approximately 38%, while an 11 mm skull reduced it to around 10%. However, the study had notable methodological limitations, including variability in phantom fabrication that led to inconsistent rSO_2_ readings despite having the same ink concentrations. Additionally, the authors concluded that rSO_2_ values are reliable when skull thickness is below 8 mm, a claim that contradicts their own findings, which showed greater error with thinner skulls.

Findings from studies on skull thickness remain somewhat contradictory and inconclusive, as one study suggest thicker skulls impede light transmission, while the other reports greater accuracy with increased skull thickness. Table 4 provides a summary of these studies, outlining publication information, optical method utilised, phantom characteristics and main outcomes.

### 4.5. Pathologies

In the subject of pathologies, two studies on oedema were found. Brain oedema is a common response to acute central nervous system injuries, such as trauma, tumours, infections, and ischemia [27]. It refers to the abnormal accumulation of excess water within brain tissue [27]. When patients experience brain oedema, the water content increases either inside or outside the brain cells. If the water content increases inside the cells, the cells will expand. In contrast, when water accumulates outside the cells, the density of the cells decreases [28]. These changes in cell size and density alter the scattering properties of brain tissue. Additionally, the thickness of the CSF layer can also increase during brain oedema [28].

**Figure 11:**
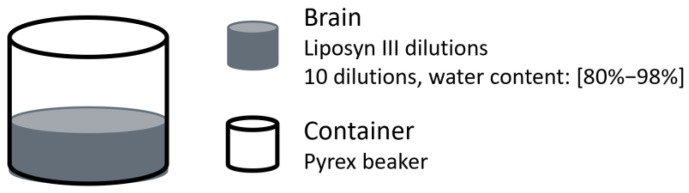
Brain oedema representation from the study by Johnson et al. [27]. Ten brain phantoms were prepared by diluting Liposyn III to achieve water contents from 80% to 98%.

In 1997 Johnson et al. examined the relationship between water content and absorption differences at NIR wavelengths using a Pacific Scientific MKII-6350 NIR spectrophotometer [27]. Chemical phantoms were prepared by diluting Liposyn III, with water content ranging from 80% to 98% (Figure 11). The results identified the strongest absorption band within the 960–970 nm range. Additionally, the absorption difference between two selected wavelengths, specifically the absorption at 957 nm minus that at 703 nm, was correlated to the water percentage in the solution, achieving a correlation coefficient of R^2^ = 0.985 ± 0.017 based on linear regression. The authors suggested that this difference method helps minimise scattering effects due to the weak wavelength dependence of scattering.

**Figure 12:**
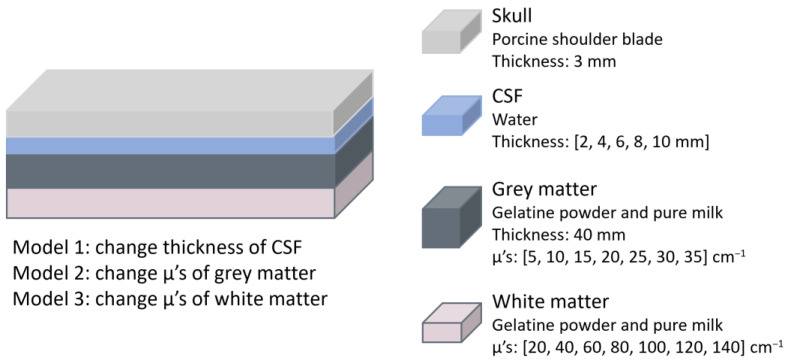
Brain oedema representation from the study by Liu et al. [28]. The materials and dimensions of each layer are shown. Three models were used to represent oedema by altering the thickness or absorption of specific layers. CSF = cerebrospinal fluid; μ’s = reduced scattering coefficient.

In 2016 Liu et al. conducted a study to determine whether NIRS could effectively detect brain oedema by assessing if this condition altered the optical signal [28]. The custom-made NIRS-based system used an LED at 760 nm and two photoelectric detectors placed at 30 and 40 mm (proximal and distal). The phantom model consisted of four layers representing the skull, CSF, grey and white matter, with the scalp excluded, as the authors suggested it had minimal influence on light intensity changes [28]. The skull was simulated using a 3 mm thick porcine shoulder blade, while a water layer represented the CSF. Grey and white matter were mimicked using a two-layer phantom composed of gelatine powder and milk (Figure 12). To simulate oedema, three experimental conditions were tested, each corresponding to changes that occur in the human brain during the condition. Model 1 varied the CSF layer thickness from 2 to 10 mm in 2 mm increments; Model 2 altered the scattering properties of grey matter by adjusting gelatine and milk concentrations; and Model 3 changed the scattering properties of white matter similarly. For Models 2 and 3, the reference values for the μ’s were obtained from a healthy individual, as the authors noted that no in vivo measurements of μ’s in brain oedema patients exist. They therefore referred to literature on traumatic brain oedema in rats to estimate the range of change.

The results showed that the detected light intensity increases as brain oedema occurs, regardless of which layer the pathology occurred in. This suggests that the system can monitor changes in brain oedema, though it cannot provide absolute values for its assessment.

**Figure 13:**
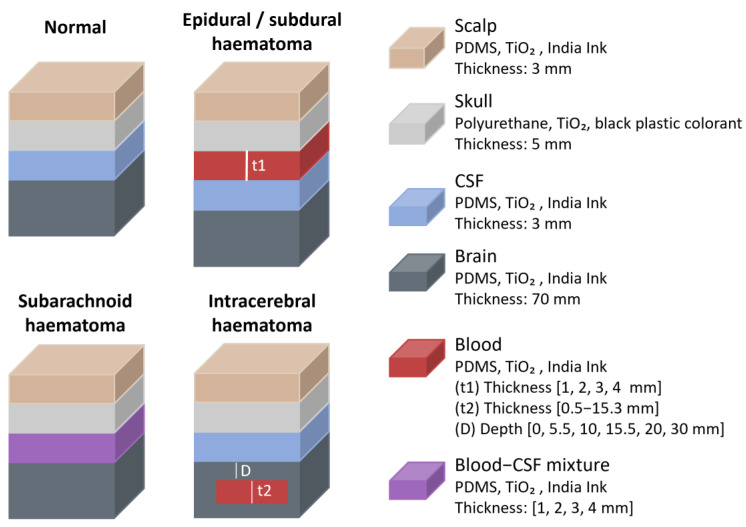
Haematomas representation from the study by J. Wang et al. [29]. Figure illustrates normal head and different types of haematomas. Materials and layer dimensions are shown. PDMS = polydimethylsiloxane; TiO_2_ = titanium dioxide; CSF = cerebrospinal fluid.

Regarding studies that focused on haematomas, in 2019 J. Wang et al. conducted in vitro measurements using a commercial NIRS-based haematoma detector, the Infrascanner (InfraScan, Inc., Philadelphia, PA, USA), to quantify the effect of haematomas on optical signals [29]. The device uses an illumination fibre emitting at 805 nm and a collection fibre, separated by 40 mm. A multilayer solid head phantom was constructed (Figure 13), consisting of scalp (3 mm), skull (5 mm), CSF (3 mm), and cerebral tissue, with each layer matched to adult human head optical properties. The scalp, CSF, and cerebral tissue were made from PDMS mixed with TiO_2_ and India Ink to adjust scattering and absorption properties, while the skull was fabricated using polyurethane mixed with TiO_2_ and black plastic colourant. Various haematoma types and thicknesses were simulated, including intracranial (epidural, subdural, and subarachnoid) and intracerebral haematomas. This approach aligns with prior CT imaging studies and is consistent with phantoms described in standards for established medical imaging modalities [29]. Epidural and subdural haematomas were modelled by placing a blood-simulating layer between the skull and CSF, while subarachnoid haematomas were represented by a CSF-diluted blood layer between the skull and cerebral tissue. Intracerebral haematomas were simulated using embedded discs within the cerebral tissue. Absorption changes were calculated by comparing normal phantoms with those containing haematomas.

The study revealed that haematomas influenced NIR absorbance based on their presence, depth, and thickness. Sensitivity to superficial haematomas (epidural, subdural and subarachnoid) was high. For intracerebral haematomas, light attenuation decreased strongly with depth and increased to a smaller degree with haematoma thickness. The maximum depth at which haematomas remained detectable was approximately 2.5 cm below the phantom surface, suggesting that deeper haematomas would likely have no measurable effect on the optical signal, at least while using this particular device, as factors like source–detector separation, wavelength, and source type could potentially alter detection depth.

**Figure 14:**
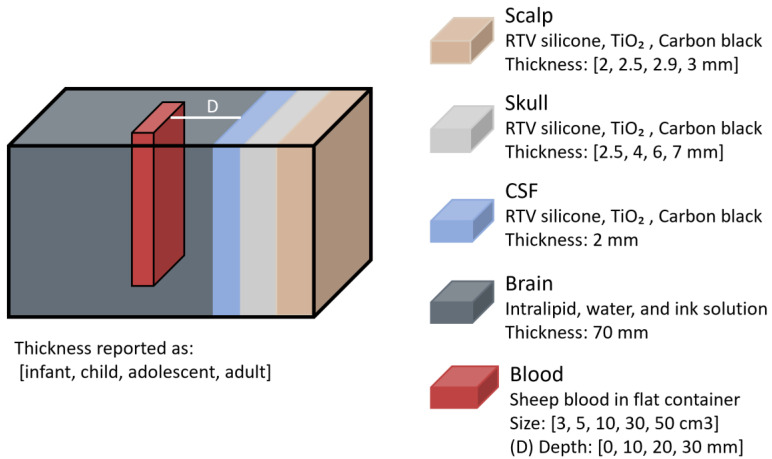
Haematoma representation from the study by L. Wang et al. [30]. Phantom layers were designed with different thicknesses corresponding to infant, child, adolescent, and adult heads. RTV = room-temperature-vulcanizing; TiO_2_ = titanium dioxide; CSF = cerebrospinal fluid.

Another study on haematomas, conducted by L. Wang et al. in 2022 [30], examined how NIR light propagates through head models of different age groups and how intracranial haematomas of varying sizes and depths influence optical density (OD) [30]. Phantoms representing infant, child, adolescent, and young adult heads were constructed with solid scalp, skull, and CSF layers made of room-temperature-vulcanizing (RTV) silicone, TiO_2_, and Carbon black, while brain tissue was simulated using an intralipid, water, and ink solution (Figure 14). According to the authors, the optical properties of each layer were matched to values reported in prior in vitro studies. Haematomas of different volumes (3, 5, 10, 30, and 50 cm^3^) were modelled using sheep whole blood and placed at varying depths (0, 10, 20, and 30 mm) from the CSF layer. The same NIRS-based haematoma detector (Infrascanner) as in the previous study was used to measure OD differences between healthy and haematoma phantoms.

Findings revealed that haematomas had a stronger effect on light propagation in younger head models, likely due to their thinner superficial layers, leading to greater OD changes. The impact of the haematomas was most pronounced when they were closer to the surface, with OD differences decreasing as lesion depth increased. Additionally, larger haematoma volumes resulted in greater OD changes.

All the studies assessing pathologies as a confounding variable used NIRS-based technology, with no studies using other optical techniques. Both studies on oedema conclude that water content influences NIR signals, while the two studies on haematomas confirm their impact on NIR signals, with effects varying by size, depth, and phantom characteristics. However, none of the studies examined whether these effects could interfere with cerebral parameter estimation, for example, they did not determine if increased water content or haematoma presence could be misinterpreted as elevated oxygenation. A summary of the research on pathologies is presented in Table 5, where the pathology, optical method used, phantom characteristics, and main results are shown.

## 5. Discussion

### 5.1. Research Directions

Firstly, most of the current research is device-specific, analysing parameters derived from proprietary algorithms rather than assessing direct light-tissue interaction. Light tissue interaction refers to the way light is absorbed, scattered, and reflected by tissues, influenced by factors such as tissue composition, thickness, and optical properties. This interaction forms the fundamental basis for accurate optical measurements. However, by focusing on proprietary algorithms and device-specific designs, there is a limitation in applicability across devices. For example, findings from one commercial oximeter may not translate to another because of factors like source–detector distance and sensor reflectivity, as demonstrated by Afshari et al. [1,3]. Thus, there is a need for a comprehensive analysis of each confounder’s effect on the optical signal itself.

Secondly, phantoms used to study confounders compromise on geometry, commonly reducing head shape to a box (e.g., liquid-filled containers) or rectangular tissue slabs. Only one study employed a more realistic skull geometry, combining in vitro and ex vivo methods, though it relied on calf skulls with the brain removed to accommodate a simple phantom [25]. There is a clear gap in the use of anatomically accurate phantoms that closely replicate human head structures.

Moreover, anatomical layers in phantoms are often oversimplified, with some studies completely omitting layers like scalp or skull, suggesting they are irrelevant despite evidence of their influence, or combining distinct layers with different optical properties into one. For example, some models represent skin, scalp, and skull as a single layer, resulting in oversimplified two-layer models.

Only five studies compared their findings to clinical data [1,3,22,25,29], although two of these used only animal models [22,25]. Some authors [22] suggested that pigs have anatomy similar to humans, which may explain agreement with their results, while others reported contrasting findings, noting that cats, rats, and piglets have much thinner skull bones compared to the calf bones used in their study [25]. In contrast, studies validating their findings against human data showed good agreement with phantom experiments [1,3,29]. Nevertheless, additional clinical research is still needed, ensuring that measurements of confounders, such as skin pigmentation, are performed objectively and quantitatively rather than using subjective scales [1].

Finally, most studies focus on NIRS, while only a few have explored DCS, and none have investigated PPG. As NIRS primarily focuses on the DC component of the optical signal, three main types of phantoms are commonly used: (1) solid models using materials like silicone or gelatine to simulate liquids like CSF or blood, (2) liquid phantoms where the liquid remains static, and (3) phantoms with circulating liquid (achieved via pumps) but with rigid walls that prevent the volume fluctuations typical of biological vessels. Notably, there is a complete lack of phantoms capable of producing pulsatile signals.

This limitation is especially important, as PPG might be a promising tool for assessing cerebral parameters like ICP [5,31,32,33]. PPG phantoms require a distensible vessel or channel wall to produce a pulsatile signal that reflects volumetric changes, a feature all current models lack. There is a clear need for a tool that allows comprehensive in vitro assessment of confounders in optical brain monitoring. Such tool should address existing gaps, including oversimplified geometry, limited anatomical layers, and the inability to produce pulsatile signals.

By developing more advanced phantom models that can simulate real-life physiological conditions, such as pulsatile signals, realistic anatomical structures, and accurate optical tissue properties, researchers can gather more quantitative data on the effects of confounders in optical brain monitoring and assess their impact on the optical signal itself, beyond the parameters estimated of specific commercial devices.

### 5.2. Limitations

The search was limited to three databases (Scopus, PubMed, and IEEE Xplore), potentially excluding relevant studies published in other repositories, and only English language studies were included, research published in other languages was omitted. Additionally, there is a potential risk of bias in study selection. The study focused specifically on certain confounders (extracerebral layers, skin pigmentation, skull thickness, and specific pathologies), potentially overlooking other relevant confounders that may influence optical brain monitoring. The variation in phantom models and optical techniques across studies introduced heterogeneity that restricts direct comparisons of results. This, as well as the limited number of available studies, impedes definitive conclusions.

Additional research is required to confirm the effects of confounders in brain optical monitoring, ideally using anatomically realistic phantoms that replicate tissue layers, geometry, and pulsatile signals, and that allow direct assessment of light-tissue interactions beyond device-specific algorithms.

## 6. Conclusions

This paper has reviewed the state of the art on the in vitro evaluation of confounders in brain optical monitoring, including the effects of extracerebral layers, skin pigmentation, skull thickness, and pathologies like oedema and haematomas. The main characteristics of the phantom models and key findings from existing studies have been discussed, highlighting both agreements and discrepancies between studies.

Current in vitro models are usually evaluated using NIRS, with limited exploration of alternative optical techniques like DCS and no studies using PPG. Given the limitations of existing research, future studies should move beyond the constraints of specific commercial devices, incorporate more anatomically realistic models, and avoid oversimplified geometries and limited anatomical layers. Additionally, the development of phantoms capable of generating pulsatile signals is essential for the evaluation of PPG-based monitoring. Ultimately, this will lead to a better understanding of how confounding variables affect the estimation of cerebral parameters and improve the reliability of optical brain monitoring techniques.

## Figures and Tables

**Table 1:** Keywords used in the systematic search of articles.

In Vitro Approach	Location	Optical Technique	Confounder
In vitro	Brain	Optical	Extracerebral
Phantom	Head	Optics	Extracranial
	Cerebral	NIRS	Skin pigmentation
		Near-Infrared Spectroscopy	Skull thickness
		DCS	Skull density
		Diffuse Correlation Spectroscopy	Edema
		PPG	Oedema
		Photoplethysmography	Haemorrhages
			Hemorrhages

**Table 2:** Studies assessing extracerebral layers as a confounding variable in optical brain monitoring. NIRS = near-infrared spectroscopy; fd = frequency-domain. CW = continuous-wave; TR = time-resolved; DCS = diffuse correlation spectroscopy; FD-DOS = frequency-domain diffuse optical spectroscopy PDMS = polydimethylsiloxane; TiO_2_ = titanium dioxide; CSF = cerebrospinal fluid; SO_2_ = brain oxygen saturation.

Author	Year	Optical Technique	Phantom Main Characteristics	Results
Afshari et al. [3]	2019	NIRS oximeters (Fore-Sight Elite, SenSmart X-100)	Brain: 3-D printed cerebrovascular module with an array of channels. CSF: PDMS and TiO_2_. Extracerebral layer: PDMS, TiO_2_ and India ink. Blood: bovine blood with sodium dithionite to induce desaturation.	Increasing superficial layer thickness caused a consistent decrease in accuracy and sensitivity for the Fore-Sight Elite device. SenSmart presented less variations.
Kurth and Thayer [20]	1999	fdNIRS	Brain: plastic cylinder with a vascular network. Extracerebral layer: clear polyester resin with TiO_2_ and embedded vascular network. Three different thicknesses (6, 10 and 20 mm). Human blood: oxygen levels adjusted by introducing O_2_ and N_2_.	Extracerebral layer thickness affected the accuracy. No significant impact was observed with 6 mm and 10 mm layers, but the 20 mm layer led to inaccuracies in SO_2_ estimation of more than 20%.
Zhang et al. [21]	2019	CW-NIRS	Five-layer solid phantom mimicking scalp, skull, CSF, grey matter, and white matter. Layers made of agar, Intralipid solution, and India Ink. Absorption coefficient of grey matter was varied to simulate a change in brain activity.	The presence of extracerebral layers led to underestimation of grey matter absorption changes.
Verdecchia et al. [22]	2016	DCS	Box made of dark polyvinyl chloride. A polyester Mylar sheet was used to divide the phantom in two layers. Both layers contained Intralipid solution. Methyl cellulose was added progressively to the bottom layer to increase viscosity.	The model was able to separate scalp and brain flow, but the error was larger as top-layer thickness increased, reaching up to ~40% at 10 mm thickness.
Verdecchia et al. [23]	2015	Hybrid DCS and TR-NIRS	Two-layer Intralipid phantom. Bottom layer was connected to a peristaltic pump and had glass marbles to randomise flow direction. Flow rate was varied, and India ink was added to change absorption in one of the layers.	Extracerebral thickness influenced system sensitivity to absorption and flow changes in the bottom layer, with the effect varying by source–detector distance. Depth sensitivity increased with greater source–detector separation.
Forti et al. [24]	2023	Hybrid DCS and FD-DOS	Black acrylic aquarium with a plastic film attached to separate in two layers. Both layers contained Intralipid solution. Ink was added to the second layer to increase absorption.	Estimated flow indices for the first layer were relatively unaffected by second-layer changes in flow and absorption, but flow estimation errors could not be determined.

**Table 3:** Studies assessing skin pigmentation as a confounding variable in optical brain monitoring. NIRS = near-infrared spectroscopy; CSF = cerebrospinal fluid; PDMS = polydimethylsiloxane; TiO_2_ = titanium dioxide.

Author	Year	Optical Technique	Phantom Main Characteristics	Results
Afshari et al. [1]	2022	NIRS oximeters (Fore-Sight Elite and SenSmart X-100)	Brain: 3-D printed cerebrovascular module with an array of channels. CSF: PDMS and TiO_2_. Extracerebral layer: PDMS, TiO_2_ and India ink. Skin: PDMS, TiO_2_ and water-soluble nigrosine in different concentrations to change pigmentation. Blood: bovine blood with sodium dithionite to induce desaturation.	An increase in melanin content led to an underestimation of saturation levels, with the effect being most pronounced at low saturation values. Sensors with low reflectivity and larger source–detector distances showed better accuracy with different skin pigmentations.
Pringle et al. [25]	1998	NIRS: oximeter (NIRO)	Brain phantom consisted of a condom filled with calf blood, deoxygenated using yeast. Calf skin and skull with varying thickness amongst the 16 calves.	There were no detectable differences between black and white calf skin. The authors suggested that skin pigmentation may not have been fully considered as a factor in the study.

**Table 4:** Studies assessing skull thickness as a confounding variable in optical brain monitoring. NIRS = near-infrared spectroscopy; rSO_2_ = regional oxygen saturation.

Author	Year	Optical Technique	Phantom Main Characteristics	Results
Pringle et al. [25]	1998	NIRS: commercial oximeter (NIRO)	Brain phantom consisted of a condom filled with calf blood, deoxygenated using yeast. Calf skin and skull with varying thickness amongst the 16 calves.	All except the two thickest skulls (13 and 14 mm) allowed sufficiently strong NIRS signals to accurately detect changes in haemoglobin oxygenation.
Leibuss et al. [26]	2021	NIRS: commercial oximeter (INVOS)	Brain and skull made with gelatine, Intralipid water solution and ink. Six different skull thickness.	The difference between rSO_2_ values from the brain alone and from the brain with overlying skull and skin decreased as skull thickness increased.

**Table 5:** Studies assessing pathologies as a confounding variable in optical brain monitoring. NIRS = near-infrared spectroscopy; CSF = cerebrospinal fluid; PDMS = polydimethylsiloxane; TiO_2_ = titanium dioxide; RTV = room-temperature-vulcanizing.

Author	Year	Pathology	Optical Technique	Phantom Main Characteristics	Results
Johnson et al. [27]	1997	Oedema	NIRS	Chemical phantoms made of diluted Liposyn III, with water content ranging from 80% to 98%.	The water percentage in the solution was correlated to the absorption difference between two selected wavelengths (957 nm and 703 nm), with correlation coefficient R^2^ = 0.985 ± 0.017.
Liu et al. [28]	2016	Oedema	NIRS	Four-layered phantom. Skull: porcine shoulder blade. CSF: water. Grey and white matter: gelatine powder and milk. Oedema was simulated by changing CSF thickness and scattering properties in the grey and white matter.	Detected light intensity increases with oedema, regardless of the affected layer.
J. Wang et al. [29]	2019	Haematoma	NIRS	Scalp, CSF, cerebral tissue, and haematomas: PDMS with TiO_2_ and India Ink. Skull: polyurethane with TiO_2_ and black plastic colourant. Haematoma: epidural, subdural, and subarachnoid and intracerebral simulated by placing layers in specific regions of the phantom.	Haematomas affect NIR absorbance, with light attenuation decreasing strongly with depth and slightly increasing with haematoma thickness. Haematomas were detectable up to a depth of 2 to 2.5 cm below the surface.
L. Wang et al. [30]	2022	Haematoma	NIRS	Scalp, skull and CSF: RTV silicone, TiO_2_ and Carbon Black. Brain: Intralipid, water, and ink solution. Haematoma: Sheep whole blood in flat containers within the brain tissue layer. Varying volumes and placed at different depths within the brain layer.	Haematomas affect the optical signal, with stronger effects in younger models due to thinner superficial layers. The impact increases with larger size and shallower depth.

## Data Availability

The original contributions presented in this study are included in the article. Further inquiries can be directed to the corresponding author.
